# A signature of hypoxia-related factors reveals functional dysregulation and robustly predicts clinical outcomes in stage I/II colorectal cancer patients

**DOI:** 10.1186/s12935-019-0964-1

**Published:** 2019-09-23

**Authors:** Yi-feng Zou, Yu-ming Rong, Ying-xin Tan, Jian Xiao, Zhao-liang Yu, Yu-feng Chen, Jia Ke, Cheng-hang Li, Xi Chen, Xiao-jian Wu, Ping Lan, Xu-tao Lin, Feng Gao

**Affiliations:** 1 Department of Colorectal Surgery, The Sixth Affiliated Hospital of Sun Yat-sen University, Guangzhou, Guangdong China; 2 Department of Medical Oncology, The Sixth Affiliated Hospital of Sun Yat-sen University, Guangzhou, Guangdong China; 3 Department of Gastrointestinal Endoscopy, The Sixth Affiliated Hospital of Sun Yat-sen University, Guangzhou, Guangdong China; 4 Guangdong Institute of Gastroenterology, Guangdong Provincial Key Laboratory of Colorectal and Pelvic Floor Diseases, The Sixth Affiliated Hospital of Sun Yat-sen University, 26 Yuancun Erheng Rd, Guangzhou, 510655 Guangdong China; 5 Department of VIP Region, Cancer Center of Sun Yat-sen University, Guangzhou, Guangdong China

**Keywords:** Hypoxia genes, Prognostic, Colorectal cancer, Prediction model

## Abstract

**Background:**

The hypoxic tumor microenvironment accelerates the invasion and migration of colorectal cancer (CRC) cells. The aim of this study was to develop and validate a hypoxia gene signature for predicting the outcome in stage I/II CRC patients that have limited therapeutic options.

**Methods:**

The hypoxic gene signature (HGS) was constructed using transcriptomic data of 309 CRC patients with complete clinical information from the CIT microarray dataset. A total of 1877 CRC patients with complete prognostic information in six independent datasets were divided into a training cohort and two validation cohorts. Univariate and multivariate analyses were conducted to evaluate the prognostic value of HGS.

**Results:**

The HGS consisted of 14 genes, and demarcated the CRC patients into the high- and low-risk groups. In all three cohorts, patients in the high-risk group had significantly worse disease free survival (DFS) compared with those in the low risk group (training cohort—HR = 4.35, 95% CI 2.30–8.23, P < 0.001; TCGA cohort—HR = 2.14, 95% CI 1.09–4.21, P = 0.024; meta-validation cohort—HR = 1.91, 95% CI 1.08–3.39, P = 0.024). Compared to Oncotype DX, HGS showed superior predictive outcome in the training cohort (C-index, 0.80 vs 0.65) and the validation cohort (C-index, 0.70 vs 0.61). Pathway analysis of the high- and low-HGS groups showed significant differences in the expression of genes involved in mTROC1, G2-M, mitosis, oxidative phosphorylation, MYC and PI3K–AKT–mTOR pathways (P < 0.005).

**Conclusion:**

Hypoxic gene signature is a satisfactory prognostic model for early stage CRC patients, and the exact biological mechanism needs to be validated further.

## Background

Colorectal cancer (CRC) is one of the most commonly diagnosed cancers worldwide, and ranks third in terms of morbidity and mortality [1]. About half of the CRC patients are at stages I/II, and more than a quarter of the early-stage patients (I–III) relapse after initial treatment [2]. Hypoxia is a common feature of solid tumors, and contributes to tumor progression and therapeutic recalcitrance. Intra-tumoral hypoxia is considered to be an indicator of poor prognosis [3, 4], and even regulates genes involved in the invasion and migration of CRC cells, which is consistent with recent reports indicating an association between lack of oxygen and distant metastasis [5–7]. Hypoxia reduces the efficacy of not only surgical resection [8], but also radiotherapy and chemotherapy [9, 10]. Only limited options are available at present for hypoxia-related targeted therapies, and there is no unequivocal evidence from clinical trials as yet regarding their efficacy, likely due to the lack of individual-based treatment [8, 11, 12]. Therefore, an accurate and non-invasive method is needed to assess tumor hypoxia. To this end, we identified a hypoxia-related gene signature (HGS) from CRC-specific transcriptomes through high-throughput expression analysis. The HGS demarcated the stage I/II CRC patients into distinct prognostic groups, and functional and pathway analyses provided new insights in the mechanism of CRC recurrence.

## Materials and methods

### Patients

The gene expression profiles of CRC tissue samples obtained from six public cohorts, including 309 CRC patients from the CIT/GSE39582 gene microarray dataset that served as the discovery cohort, were retrospectively analyzed. The two largest individual data sets—CIT/GSE39582 and The Cancer Genome Atlas (TCGA)—were used for training and independent validation. The meta-validation cohort consisted of the remaining four microarray data sets—GSE14333, GSE17536, GSE37892 and GSE33113—which were obtained from the Gene Expression Omnibus (GEO) database. All datasets are from the GPL570 platform ([HG-U133_Plus_2] Affymetrix Human Genome U133 Plus 2.0 Array). TCGA cohort data was downloaded from Broad GDAC Firehose, and the other data sets were obtained directly in their processed format from the GEO database through Bioconductor package ‘GEOquery’. Transcripts per million (TPM) of level 3 RNA-Seq data in log2 scale was applied to calibrate the gene expression levels in TCGA cohort. The ‘combat’ algorithm of the R package ‘sva’ and the z-scores were used to correct the batch effects, in order to standardize microarray data across multiple experiments and compare them independent of the original hybridization intensities. The data of 1877 CRC patients enrolled from Sep 27 to Dec 26, 2018 was also included.

### Construction and validation of HGS

To construct a prognostic HGS, annotated functional database MSigDB (version 6.2) [13–15] was used to identify a list of hypoxia-related genes with the keyword “hypoxia”, and the HGSs measured by all platforms were selected. The log-rank test was used with 1000 randomizations (80% of samples each time) to evaluate the association between each HGS and clinical outcome in the training dataset. Genes that were repeatedly significant were selected as the candidates of the hypoxia signature. To minimize the risk of over-fitting, Cox proportional hazards regression model was applied with the least absolute shrinkage and selection operator (LASSO) (glmnet, version 2.0-16). The penalty parameter was estimated by tenfold cross-validation in the training data set at 1 SE beyond the minimum partial likelihood deviance.

A time-dependent receiver operating characteristic (ROC) curve (survival ROC, version 1.0.3) at 5 years was plotted using Kaplan–Meier estimation, and used to determine the optimal HGS cutoff to separate patients in the training data set into the low-risk and high-risk groups. The HGS corresponding to the shortest distance between the ROC curve and the point representing 100% true positive rate and 0% false-positive rate was used as the cutoff value. Univariate analysis was used to evaluate the prognostic value of the HGS in stage I/II CRC patients, and in patients at all stages in the training and independent validation cohorts. In the multivariate analyses, HGS was combined with other clinical and pathological variables.

### Functional annotation and analysis

To investigate the biological characteristics of the HGS, enrichment analysis was conducted for differentially expressed genes (DEGs) between the risk groups in TCGA CRC data set using R package ‘gProfileR’. Gene Set Enrichment Analysis (GSEA) was further performed using Bioconductor package ‘HTSanalyzeR’ to predict the significant pathways [16].

### Statistical analysis

All statistical analysis was performed in R software (version 3.5.1; http://www.Rproject.org). Descriptive statistics were computed for all variables, and expressed as mean ± standard deviation (SD) or medians and interquartile ranges (IQR) for continuous factors, and as frequencies for categorical factors. Continuous values were compared using Student-t tests between different groups. Log-rank test was used to evaluate results of the univariate analysis of HGS and other clinico-pathological factors with disease free survival (DFS). Multivariate analysis was performed with the Cox proportional hazards regression model. The C-index was calculated by ‘survcomp’ (version 1.32.0). P values less than 0.5 were considered statistically significant.

## Results

### The establishment of HGS

**Fig. 1:**
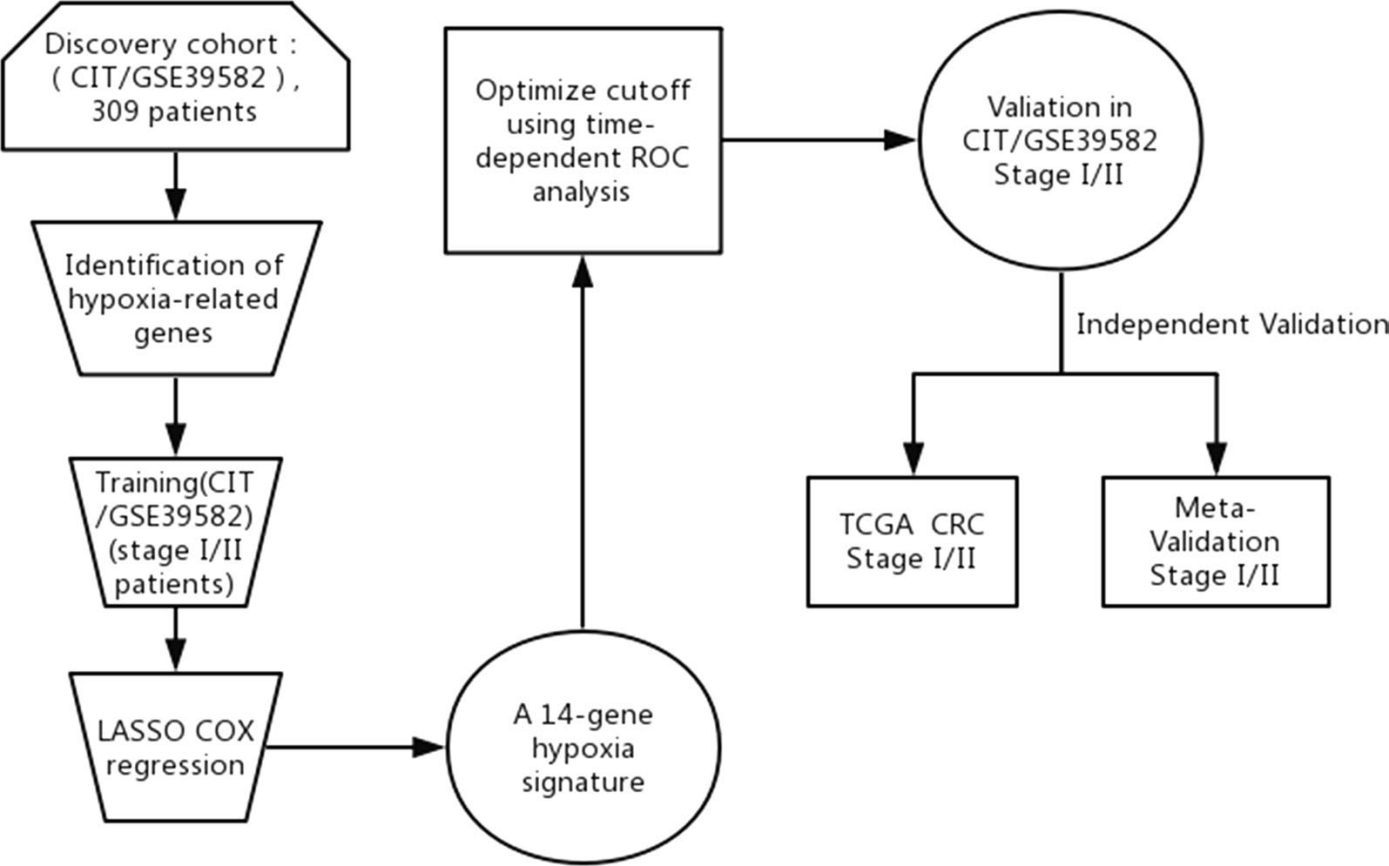
Schematic flow chart of the study procedure

**Fig. 2:**
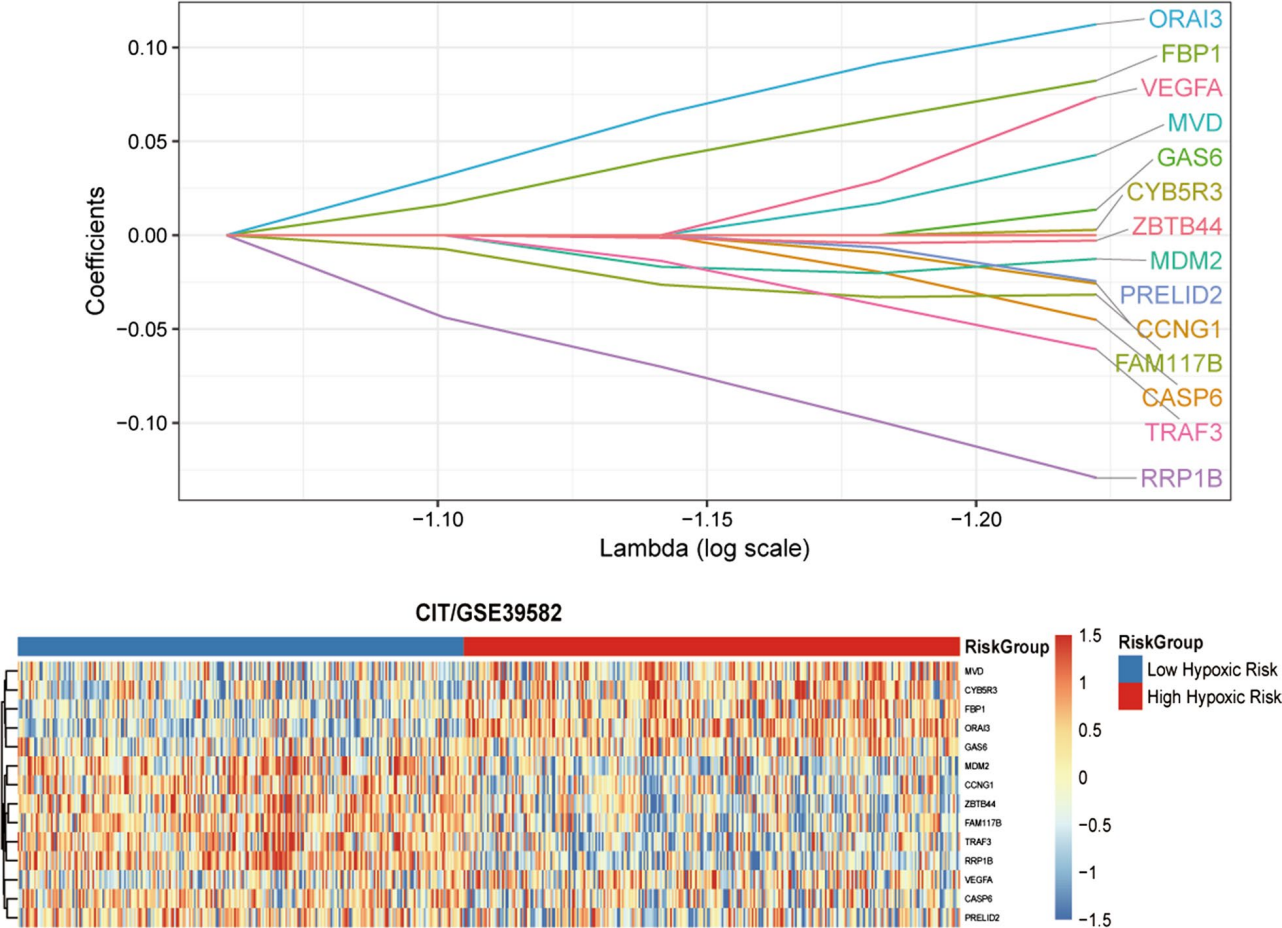
The establishment of hypoxic gene signature (HGS) using 14 hypoxia-associated genes from the LASSO COX regression

**Fig. 3:**
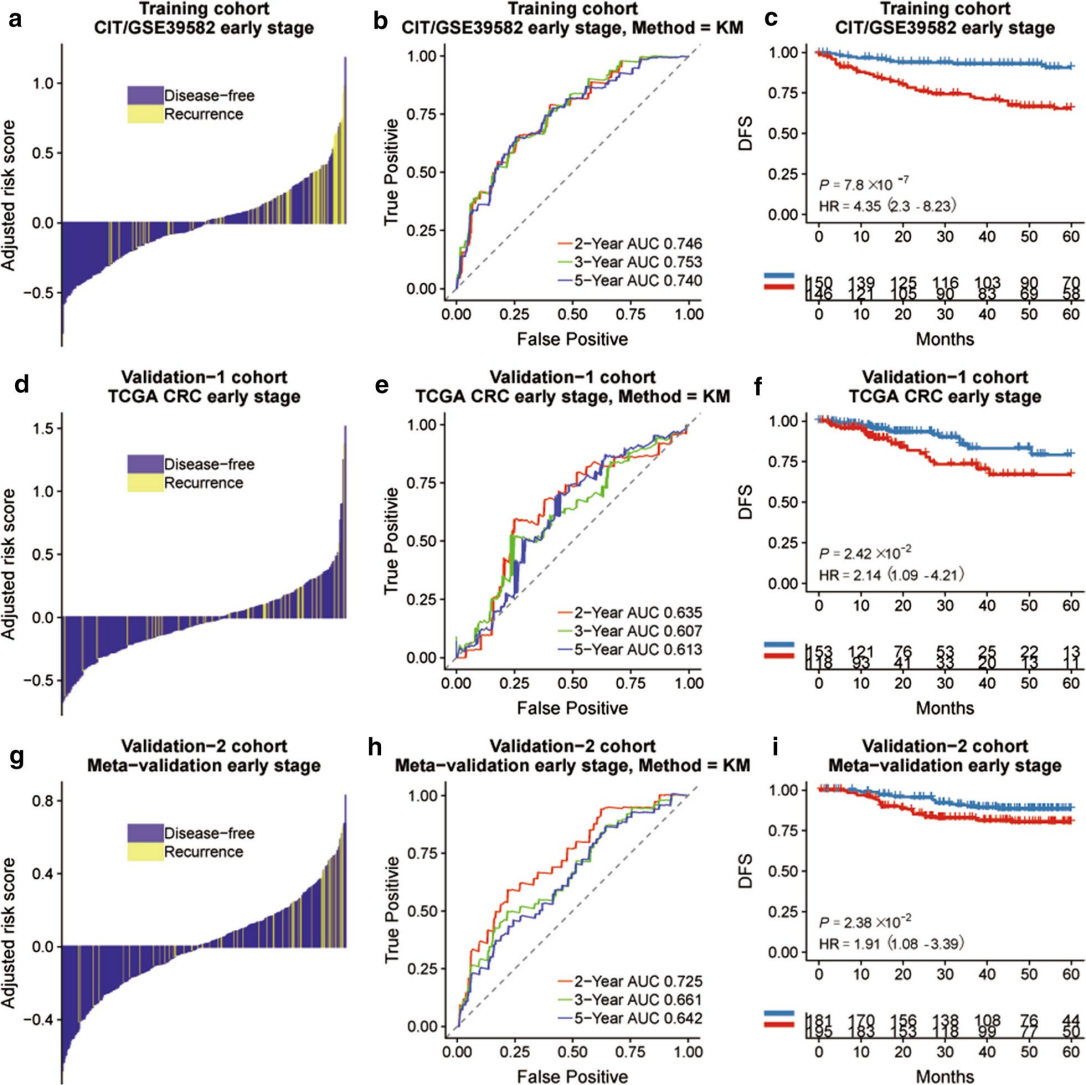
**a** Distribution of the HGS risk score in stage I/II CRC cohort and its correlation to recurrence in the training, TCGA and meta-validation cohorts, with risk scores as the continuous variable for individual patients. The DFS and recurrence in the different hypoxia risk groups of training cohort (**b**), TCGA cohort (**e**) and meta-validation cohort (**h**). Kaplan–Meier curves comparing survival of patients with low or high hypoxia risk in training cohort (**c**), TCGA cohort (**f**) and meta-validation cohort (**i**)

**Fig. 4:**
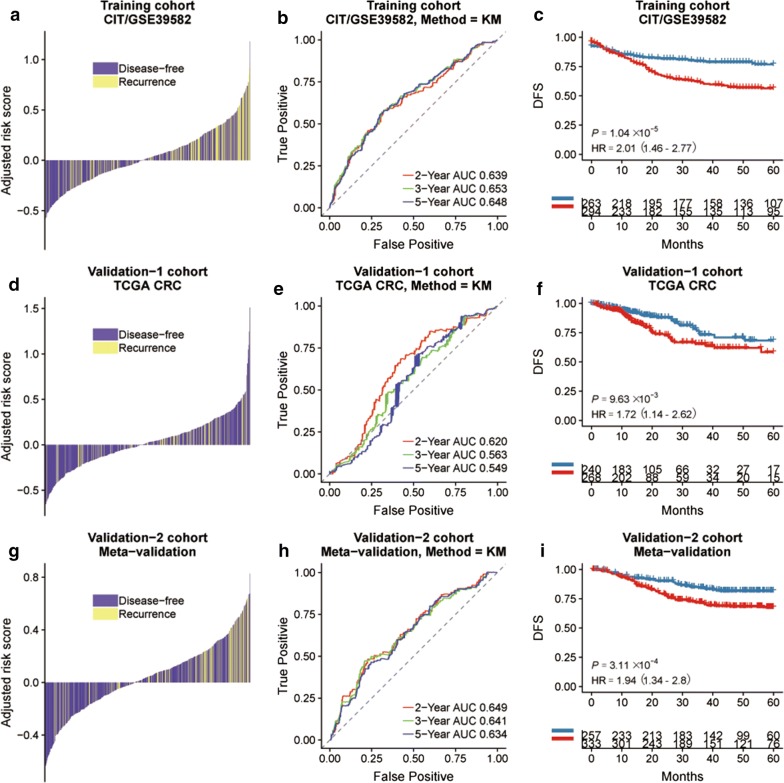
**a** Distribution of the HGS risk score and its correlation to recurrence in the training, TCGA cohort and meta-validation cohort, with risk scores as the continuous variable for individual patients. The DFS and recurrence in the different hypoxia risk groups of training cohort (**b**), TCGA cohort (**e**) and meta-validation cohort (**h**). Kaplan–Meier curves comparing survival of patients with low or high hypoxia risk in training cohort (**c**), TCGA cohort (**f**) and meta-validation cohort (**i**). P-values were calculated using log-rank tests and HR is short for hazard ratio

We analyzed the CIT gene microarray dataset (GSE39582) and created the discovery subset with 309 eligible CRC patients (Fig. 1). After exclusion of genes with MAD > 0.5 and less median expression, 1636 genes were retained for further analysis. Following selection of 80% of the repeatable genes via 1000 random Cox univariate regressions, we identified 106 genes that were associated with DFS, of which 14 hypoxia-related genes were selected to construct the HGS using LASSO Cox regression for stage I/II CRC patients (Fig. 2). The risk scores were calculate using the formula derived from the Cox model as follows: Risk score = − 0.013 × *exp*(mRNA expression level of MDM2) + 0.0733 × *exp*(mRNA expression level of VEGFA) + 0.112 × *exp*(mRNA expression level of ORAI3) + 0.043 × *exp*(mRNA expression level of MVD) − 0.060 × *exp*(mRNA expression level of TRAF3) − 0.003 × *exp*(mRNA expression level of CYB5R3) − 0.003 × *exp*(mRNA expression level of ZBTB44) − 0.045 × *exp*(mRNA expression level of CASP6) + 0.082 × *exp*(mRNA expression level of FBP1) − 0.026 × *exp*(mRNA expression level of CCNG1) − 0.032 × *exp*(mRNA expression level of FAM117B) − 0.025 × *exp*(mRNA expression level of PRELID2) − 0.129 × *exp*(mRNA expression level of RRP1B) + 0.014 × *exp*(mRNA expression level of GAS6). Based on time-dependent ROC curve analysis, the optimal cutoff of HGS for stratifying patients in the training set into the high and low risk groups was determined to be a satisfactory RFS cutoff at 5 years (Fig. 3b, e and h). The incidence of tumor recurrence was higher among the patients in the high-risk group compared to the low-risk group when the entire CIT dataset (n = 566) was used as a training cohort (Fig. 4a, P < 0.001).

### Validation of HGS

The prognostic significance of HGS was assessed with additional CRC transcription data sets that included clinical and prognostic data. Clinicopathological characteristics of three cohorts are listed in Table 1. The validation datasets consisted of TCGA datasets (n = 624) and the meta-validation cohort (n = 687), including GSE17536, GSE33113, GSE37892 and GSE14333. No significant difference was seen between the clinico-pathological features of the training and validation cohorts (Table 2). The DFS was significantly higher in the low-risk compared to the high-risk HGS group in all three cohorts (training cohort: HR = 4.35, 95% CI 2.30–8.23, P < 0.001; validation: HR = 2.14, 95% CI 1.09–4.21, P = 0.024 and meta-validation cohort: HR = 1.91, 95% CI 1.08–3.39, P = 0.024) (Fig. 3c, f and i). We compared HGS with Oncotype DX to further evaluate its prognostic value and robustness (Table 3), and found that HGS had a more optimized C-index in both training and TCGA cohorts (training cohort: 0.80 vs 0.65, TCGA cohort: 0.70 vs 0.61, Table 3). Further data mining indicated a prognostic value of HGS in all CRC cohorts (GSE39582 cohort: HR = 2.01, 95% CI 1.46–2.77, P < 0.001; TCGA cohort: HR = 1.72, 95% CI 1.14–2.62, P = 0.010; meta-validation cohort: HR = 1.94, 95% CI 1.34–2.8, P < 0.001) (Fig. 4c, f and i). Similar results were obtained in the AUC analysis (Fig. 4b, e and h).

**Table 1:** Characteristics of training, validation and meta-validation cohorts

Characteristic	TCGA	CIT/GSE39582	Meta-validation
Number of patients	624	566	687
Patients with survival data	509	557	590
Mean age, years	66.27 ± 12.76	66.85 ± 13.29	66.80 ± 12.82
Gender, n			
Male	332	310	371
Female	292	256	316
TNM stage, n			
Stage I	105	33	68
Stage II	230	264	314
Stage III	180	205	205
Stage IV	88	60	100
NA	21	4	0
CMS system, n			
CMS1	68	91	126
CMS2	207	232	252
CMS3	64	69	103
CMS4	117	127	155
NA	168	47	51
Tumor location, n			
Left	354	342	233
Right	270	224	185
NA			269
RFS event, n			
Yes	100	177	141
No	416	380	449
NA	108	9	97
OS event, n			
Yes	67	191	73
No	557	371	104
NA		4	220
DFS event, n			
Yes	146	248	188
No	386	314	434
NA	92	4	65
MMR status, n			
MSI	189	75	25
MSS	431	444	65
NA	4	47	597
CIMP status, n			
Positive		91	26
Negative		405	64
NA	624	70	597
CIN status, n			
Positive		353	
Negative		110	
NA	624	103	687
TP53 status, n			
Wild type		161	
Mutation		190	
NA	624	215	687
KRAS status, n			
Wild type	34	328	70
Mutation	30	217	20
NA	560	21	597
BRAF status, n			
Wild type	32	461	73
Mutation	3	51	17
NA	589	54	597

**Table 2:** Univariate and multivariate analysis of HGS, clinical and pathologic factors in validation cohorts

Characteristic	CIT/GSE39582 CRC				TCGA CRC				Meta-validation			
	Univariate		Multivariate		Univariate		Multivariate		Univariate		Multivariate	
	HR (95% CI)	P-value	HR (95%CI)	P-value	HR (95% CI)	P-value	HR (95% CI)	P-value	HR (95% CI)	P-value	HR (95% CI)	P-value
HGS	8.66 (4.37–17.17)	< 0.001	7.54 (3.78–15.06)	< 0.001	2.59 (1.08–6.25)	0.04	2.59 (1.08–6.25)	0.04	8.25 (3.09–22.03)	< 0.001	7.25 (2.72–19.29)	< 0.001
Age	1.01 (0.99 –1.03)	0.58			1.01 (0.98–1.04)	0.37			0.97 (0.95–1.00)	0.02	0.98 (0.96–1.00)	0.05
Gender	1.53 (0.898–2.62)	0.12			1.56 (0.81–2.99)	0.18			0.68 (0.39–1.16)	0.15		
TNM stage	7.89 (1.11–55.91)	< 0.001	5.76 (0.81–41.12)	0.08	1.83 (0.76–4.41)	0.17			3.99 (1.24–12.80)	0.01	3.73 (1.16–11.99)	0.03
Tumor location	1.08 (0.64–1.84)	0.78			1.09 (0.59–2.04)	0.78			1.30 (0.57–2.99)	0.53		
MMR status	1.63 (0.70–3.82)	0.25			0.64 (0.34–1.24)	0.18			1.27 (0.42–3.86)	0.67		
CIMP status	0.95 (0.44–2.02)	0.89							0.91 (0.32–2.55)	0.85		
CIN status	1.69 (0.75–3.81)	0.20										
TP53 mutation	1.39 (0.78–2.48)	0.27										
KRAS mutation	1.44 (0.86–2.40)	0.16			1.02 (0.23–4.60)	0.98			1.40 (0.50–3.94)	0.52		
BRAF mutation	1.42 (0.57–3.58)	0.45							1.72(0.61–4.81)	0.30

**Table 3:** C-index for hypoxic risk compared with Oncotype DX in three cohorts

Cohorts	HGS		Oncotype DX	
	C-index	95% CI	C-index	95% CI
CIT/GSE39582 (training)	0.80	0.70–0.90	0.65	0.53–0.77
TCGA (validation)	0.70	0.55–0.85	0.61	0.44–0.77
Meta-validation	0.68	0.55–0.80	0.73	0.64–0.83

### Independent influencing factors of HGS

Univariate and multivariate analyses were used to determine whether patient age, gender, tumor stage, tumor location, pathological gene status and HGS were associated with prognosis in stage I/II CRC patients. The univariate analysis showed that HGS was significantly associated with a poor outcome in the three cohorts, (GSE39582 cohort: HR = 8.66, 95% CI 4.37–17.17, P < 0.001; TCGA cohort: HR = 2.59, 95% CI 1.08–6.25, P = 0.04; and meta-validation cohort: HR = 8.25, 95% CI 3.09–22.03, P < 0.001, Table 2). After adjusting for other factors in the multivariate analysis, it remained an independent prognostic factor (GSE39582 cohort, HR = 7.54, 95% CI 3.78–15.06, P < 0.001; TCGA cohort, HR = 2.59, 95% CI 1.08–6.25, P = 0.04; and meta-validation cohort, HR = 7.25, 95% CI 2.72–19.29, P < 0.001, Table 2).

### Pathways analysis of HGS predicted risk group

**Fig. 5:**
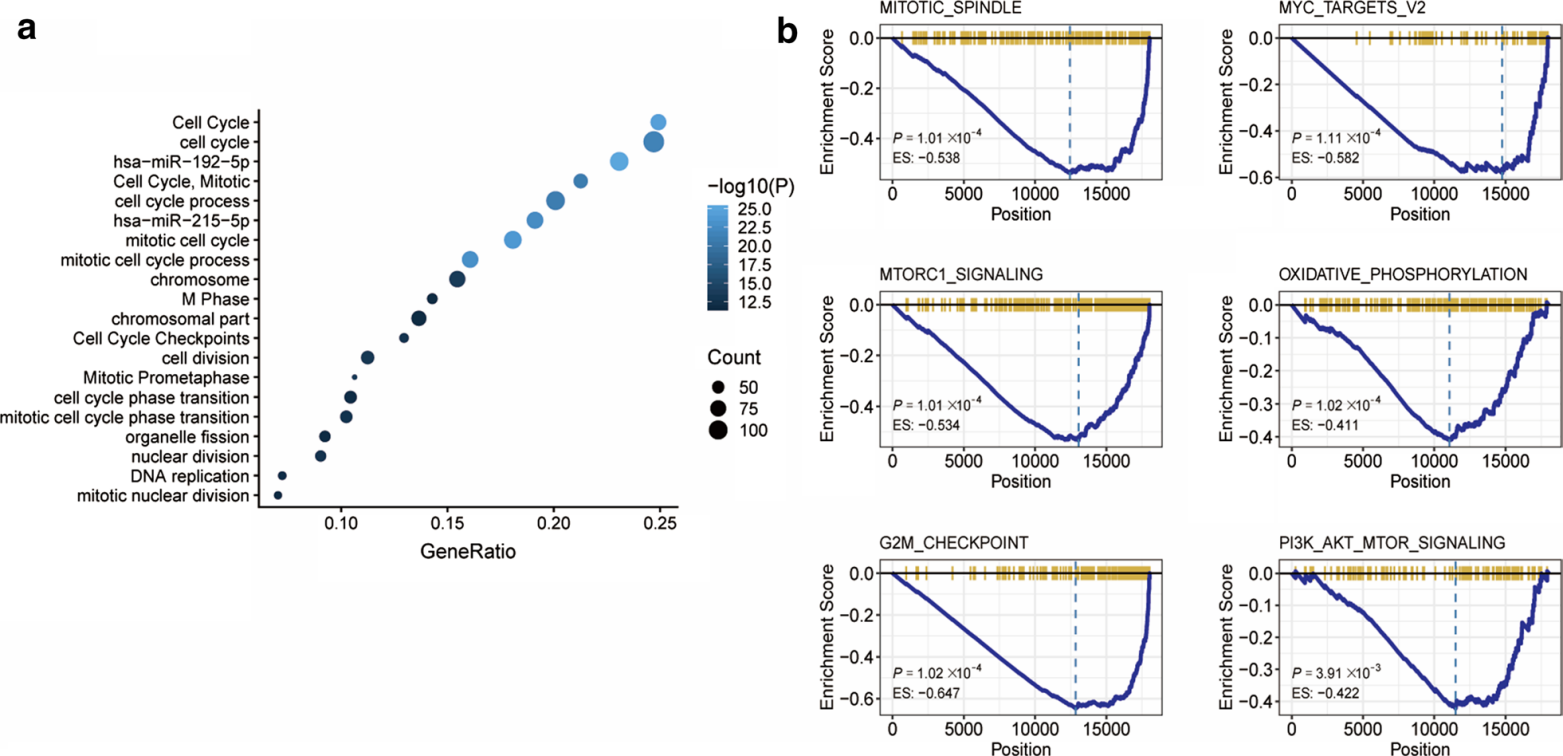
**a** Functional annotation of the HGS. Enrichment analysis of the DEGs between risk groups. **b** GSEA showed that mTROC1, G2-M, mitosis, oxidative phosphorylation, MYC and PI3K–AKT–mTOR were downregulated in high hypoxia risk patients

Gene ontology and KEGG pathway enrichment analysis of the DEGs and the GSEA showed a significant enrichment of metabolic pathways such as mTROC1 (P = 0.0001), G2-M (P = 0.0001), mitosis (P = 0.0001), oxidative phosphorylation (P = 0.0001), MYC (P = 0.0001), and PI3K–AKT–mTOR (P = 0.0039) (Fig. 5).

## Discussion

The current therapeutic modality for early stage CRC is surgical resection. Nevertheless, the recurrence rate of stage I/II CRC patients after surgery is still higher than 20% [17]. Despite identifying numerous genes that affect the recurrence and metastasis of CRC [18, 19], no prognostic gene signature has been validated so far. Effective prognostic biomarkers are therefore urgently needed to predict the DFS rate and risk of relapse after treatment in early-stage CRC patients. In this study, we developed a novel predictive hypoxia-related 14-gene signature for CRC, and validated it in multiple cohorts. The results suggest that the HGS can successfully predict the DFS of CRC patients after treatment.

Oxygen provides energy for cell growth and division, and is a key signaling molecule. The hypoxia inducible factors (HIFs) respond to changes in oxygen levels and cellular energy status, and trigger a transcriptional program [20] that mediates malignant transformation and progression. Not surprisingly, lack of oxygen and overexpression of HIF is associated with poor prognosis in cancer patients [21, 22]. Furthermore, tumor cells induce pro-angiogenic factors to vascularize the tumor in order to survive and proliferate under hypoxic condition, which are regulated along with the hypoxia-related genes [23]. In fact, HIF inhibitors also improve the efficacy of anti-angiogenesis drugs during cancer treatment [21, 22, 24]. Consistent with these previous studies, we found that hypoxia-related genes worsened CRC prognosis by affecting genes involved in the cell cycle, indicating that hypoxia-related drug targets can potentially improve CRC prognosis.

Several studies have shown an association between tumor hypoxia and poor therapeutic outcome in cancer patients. Oxygen deficiency reduces the efficacy of surgical resection and increases metastatic potential of tumors [25, 26]. The current endogenous markers of hypoxia cannot accurately monitor intra-tumor oxygen levels, which limits the efficacy of hypoxia-targeting drugs [8, 27]. The HGS stratified the stage I/II CRC patients into high- and low-risk groups that differed significantly in terms of DFS during a 5-year follow-up. The C index results of the 14-gene hypoxia signature showed its clinical superiority to Oncotype DX. This novel prognostic tool can thus identify CRC patients with highly hypoxic tumors that at risk of treatment failure, and enable clinicians to make informed decisions regarding treatment regimens. It may also help in calculating the possibility of tumor recurrence after surgery.

Several research groups have developed hypoxia-targeted therapy against solid tumors to improve patient survival, although clinical trials have not yielded satisfactory results [27–29]. Therefore, there is an urgent need for better therapeutic targets to improve the prognosis of CRC patients. We observed a significant enrichment of cell cycle/metabolism-related genes and functions, such as mTROC1, E2F, G2-M, mitosis, oxidative phosphorylation, MYC and PI3K–AKT–mTOR (P < 0.005), in the high-risk, low DFS group. Previous studies have found a correlation between these targets and CRC development, although they did not link these targets to tumor hypoxia [30–35]. Further studies are needed to clarify the effects of hypoxia on cell cycle in order to identify more targets and improve the prognosis of early stage CRC patients.

In conclusion, we identified a prognostic hypoxia-associated gene signature using genome-wide analysis to predict DFS in patients with stage I/II CRC. These hypoxia-associated DEGs are potential therapeutic targets against CRC. However, our study is beset with the limitations associated with all retrospective studies, in addition to systematic errors resulting from analyzing samples from disparate databases. Therefore, further clinical and pharmacological tests are needed to validate our results.

## Conclusions

We developed a novel HGS to stratify stage I and II CRC patients into high- and low-risk groups with greater accuracy compared to the currently used clinicopathological risk factors. A “risk prediction model” was also constructed using the HGS, the scores of which can be readily applied to independent prospective cohorts. HGS is a highly promising prognostic tool for personalized treatment regimens and clinical management of stage I/II CRC patients.

## Data Availability

TCGA cohort data was downloaded from Broad GDAC Firehose (http://gdac.broadinstitute.org/). The datasets generated and analyzed during the current study are available in the GSE39582 (https://www.ncbi.nlm.nih.gov/geo/query/acc.cgi?acc=GSE39582), TCGA (https://www.cancer.gov/about-nci/organization/ccg/research/structural-genomics/tcga), GSE14333 (https://www.ncbi.nlm.nih.gov/geo/query/acc.cgi?acc=GSE14333), GSE17536 (https://www.ncbi.nlm.nih.gov/geo/query/acc.cgi?acc=GSE17536), GSE37892 (https://www.ncbi.nlm.nih.gov/geo/query/acc.cgi?acc=GSE37892) and GSE33113 (https://www.ncbi.nlm.nih.gov/geo/query/acc.cgi?acc=GSE33113).

## Abbreviations

CRC: colorectal cancer
HGS: hypoxic gene signature
TCGA: The Cancer Genome Atlas
GEO: Gene Expression Omnibus
TPM: Transcripts per million
ROC: receiver operating characteristic
DEGs: differentially expressed genes
GSEA: Gene Set Enrichment Analysis
SD: standard deviation
IQR: interquartile ranges
DFS: disease free survival
HIFs: hypoxia inducible factors
